# Supplementary material for: β Oscillations of Dorsal STN as a Potential Biomarker in Parkinson’s Disease Motor Subtypes: An Exploratory Study
Source: Brain Sci. 2023 Apr 28;13(5):737. doi: 10.3390/brainsci13050737 (PMC10216185; doi:10.3390/brainsci13050737)
Supplement: Supplementary file 1 [file brainsci-13-00737-s001.zip › brainsci-2318900-supplementary.pdf]

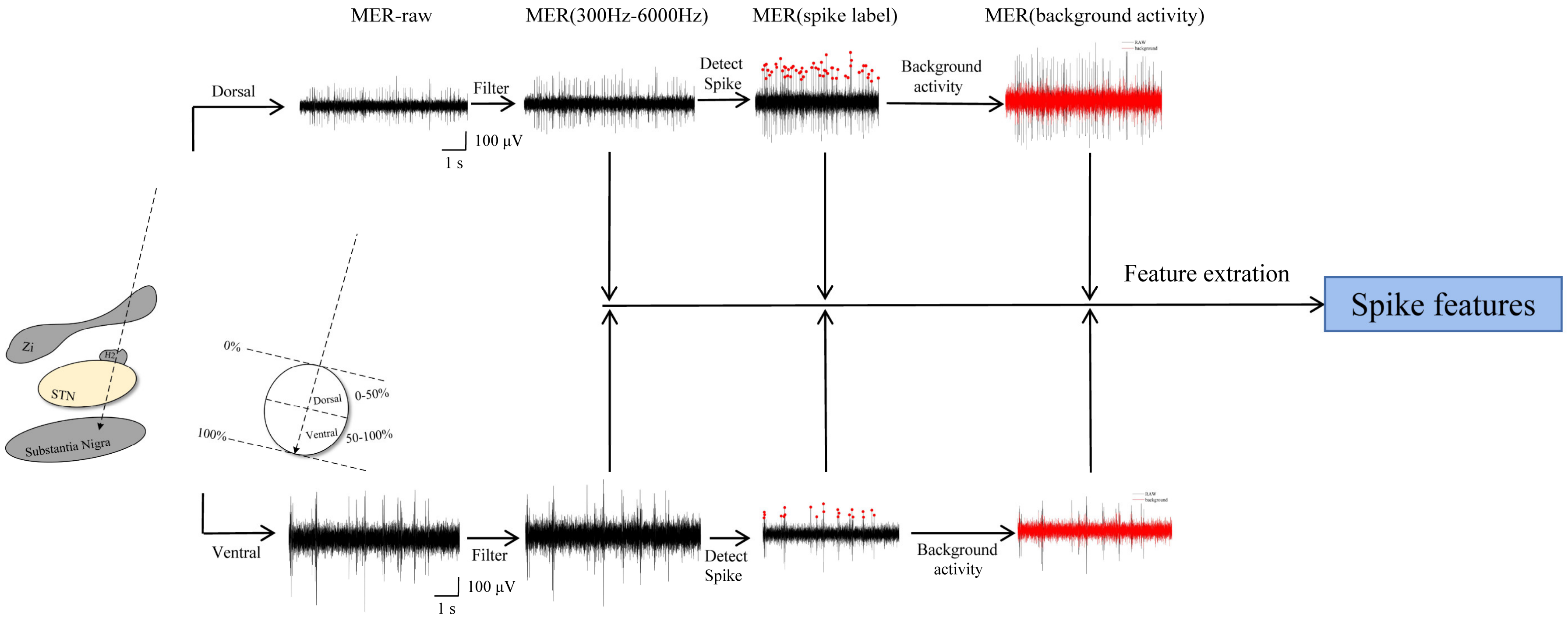

Figure S1. MER feature extraction flow chart

Table S1 Comparison of three neural markers in the dorsal and ventral STN regions between the PIGD and TD groups. Data are presented as mean (95% confidence interval).

	PIGD group (*n* = 14)	TD group (*n* = 9)	*P* value
FR Dorsal	37.45±11.71	36.56±8.30	0.84[b]
FR Ventral	36.78±12.29	35.34±6.99	0.75[b]
P-value	0.88[b]	0.74[b]	
MBI Dorsal	1.56±0.74	1.67±1.44	0.81[b]
MBI Ventral	1.94±1.59	1.73±1.53	0.75[b]
P-value	0.43[b]	0.93[b]	
AI Dorsal	0.25±0.08	0.24±0.07	0.88[b]
AI Ventral	0.29±0.07	0.29±0.09	0.96[b]
P-value	0.17[b]	0.26[b]	

FR, firing rate; MBI, modified burst index; AI, asymmetry index;
Differences between the two groups were analyzed using the *t* test[b]

Table S2 Results of the correlation analysis between MER characteristics and clinical characteristics in the PIGD group (Med-off).

MER features	H-Y	UPDRS III (Med-off)					
		UPDRS III	Tremor	Rigidity	Bradykinesia	Axial symptoms	Gait
Power_Theta	-0.162/0.579	-0.231/0.427	-0.452/0.104	-0.34/0.234	-0.388/0.17	0.02/0.946	-0.009/0.975
Power_Alpha	0.296/0.304	-0.057/0.846	-0.086/0.769	0.082/0.78	-0.133/0.65	-0.018/0.952	-0.172/0.557
Power_Beta	0.482/0.081	0.002/0.994	0.217/0.456	0.214/0.463	-0.1/0.734	-0.183/0.531	-0.149/0.612
Power_Gamma	0.116/0.693	0.073/0.805	-0.188/0.519	0.176/0.548	0.024/0.934	0.011/0.97	-0.2/0.494
Power_Dorsal_Theta	-0.09/0.759	-0.081/0.782	-0.235/0.419	-0.24/0.408	-0.162/0.58	0.156/0.594	0.334/0.243
Power_Dorsal_Alpha	-0.018/0.951	-0.326/0.256	-0.295/0.306	0.182/0.532	-0.493/0.073	-0.004/0.988	0.162/0.579
Power_Dorsal_Beta	0.116/0.693	-0.273/0.401	0.297/0.345	0.178/0.603	-0.315/0.272	-0.433/0.122	-0.214/0.464
Power_Dorsal_Gamma	0.054/0.854	0.099/0.736	-0.206/0.479	0.189/0.517	0.033/0.91	0.078/0.791	-0.074/0.801
Power_Ventral_Theta	-0.327/0.253	-0.11/0.708	-0.346/0.226	0.258/0.373	-0.249/0.391	0.105/0.721	-0.167/0.568
Power_Ventral_Alpha	0.24/0.409	0.125/0.669	0.035/0.904	0.138/0.638	0.007/0.982	0.109/0.71	-0.195/0.504
Power_Ventral_Beta	**.575/0.032***	-0.029/0.923	0.049/0.868	0.26/0.369	-0.064/0.827	-0.252/0.384	-0.19/0.515
Power_Ventral_Gamma	0.245/0.399	-0.015/0.958	-0.024/0.934	0.049/0.868	-0.055/0.851	-0.109/0.71	-0.325/0.257
Firing rate	0.08/0.786	-0.119/0.686	0.144/0.623	-0.129/0.66	-0.027/0.928	-0.246/0.397	-0.097/0.74
Asymmetry_Index	0.013/0.965	-0.262/0.366	-0.153/0.602	0.138/0.638	-0.198/0.498	-0.223/0.443	0.005/0.987
MBI	-0.332/0.246	0.057/0.846	0.324/0.259	0.196/0.502	0.036/0.904	0.185/0.526	0.237/0.415

Values are presented as rho (ρ)/P value. *Represented a statistical correlation ($P < 0.05$). MBI, modified burst index; H&Y, Hoehn and Yahr stages, UPDRS, Unified Parkinson's Disease Rating Scale.

Table S3 Results of the correlation analysis between MER characteristics and clinical characteristics in the TD group (Med-off).

MER features	H-Y	UPDRS III (Med-off)					
		UPDRS III	Tremor	Rigidity	Bradykinesia	Axial symptoms	Gait
Power_Theta	-0.156/0.689	-0.159/0.683	0.251/0.515	0.017/0.965	-0.552/0.123	0.06/0.879	-0.33/0.385
Power_Alpha	-0.092/0.814	-0.126/0.748	0.393/0.295	0.156/0.68	**-.695/0.038***	0.221/0.567	-0.266/0.489
Power_Beta	-0.294/0.443	-0.502/0.168	0.192/0.62	0.494/0.177	**-.778/0.014***	0.009/0.983	-0.422/0.258
Power_Gamma	-0.128/0.742	-0.628/0.07	0.059/0.881	0.502/0.168	**-.778/0.014***	-0.043/0.913	-0.395/0.293
Power_Dorsal_Theta	-0.092/0.814	-0.126/0.748	0.393/0.295	0.351/0.233	**-.695/0.038***	0.221/0.567	-0.266/0.489
Power_Dorsal_Alpha	-0.156/0.689	-0.092/0.814	0.351/0.354	0.017/0.965	-0.611/0.081	0.17/0.661	-0.266/0.489
Power_Dorsal_Beta	-0.138/0.724	**-.678/0.045***	-0.075/0.847	0.545/0.129	**-.770/0.015***	0.009/0.953	-0.284/0.458
Power_Dorsal_Gamma	-0.229/0.553	-0.603/0.086	-0.184/0.635	0.579/0.102	-0.151/0.699	-0.375/0.321	-0.339/0.371
Power_Ventral_Theta	-0.367/0.331	-0.201/0.604	0.05/0.898	0.153/0.694	-0.109/0.781	-0.383/0.309	-0.477/0.194
Power_Ventral_Alpha	0.119/0.76	0.343/0.366	0.46/0.213	0.128/0.743	-0.31/0.417	0.068/0.862	-0.202/0.603
Power_Ventral_Beta	0.193/0.619	0.042/0.915	0.594/0.092	0.026/0.948	-0.36/0.342	0.434/0.243	0.119/0.76
Power_Ventral_Gamma	0.009/0.981	-0.351/0.354	0.326/0.391	0.272/0.478	**-.820/0.007****	0.358/0.345	-0.092/0.814
Firing rate	0.064/0.87	-0.192/0.62	0.343/0.366	0.451/0.223	0.067/0.864	-0.477/0.194	0.266/0.489
Asymmetry_Index	-0.395/0.293	0.326/0.391	0.435/0.242	0.026/0.948	0.167/0.667	0.255/0.507	0.284/0.458
MBI	-0.156/0.689	0.318/0.404	0.1/0.797	0.179/0.645	**.720/0.029***	-0.4/0.286	-0.009/0.981

Values are presented as rho (ρ)/P value. *Represented a statistical correlation (P < 0.05). MBI, modified burst index; H&Y, Hoehn and Yahr stages; UPDRS, Unified Parkinson's Disease Rating Scale.

Table S4 Results of the correlation analysis between MER characteristics and clinical characteristics in the PIGD group (DBS-on).

MER features	UPDRS III (DBS-on)					
	UPDRS III	Tremor	Rigidity	Bradykinesia	Axial symptoms	Gait
Power_Theta	0.307/0.286	-0.128/0.664	-0.104/0.724	0.228/0.433	0.029/0.921	0.257/0.375
Power_Alpha	0.007/0.982	0.313/0.275	-0.079/0.79	0.003/0.993	-0.038/0.897	0.087/0.767
Power_Beta	-0.249/0.39	0.039/0.895	-0.185/0.527	-0.202/0.488	-0.284/0.325	-0.195/0.504
Power_Gamma	0.203/0.487	-0.183/0.531	0.06/0.838	0.187/0.522	0.038/0.897	0.257/0.375
Power_Dorsal_Theta	0.39/0.168	-0.291/0.313	0.224/0.441	0.182/0.534	0.331/0.248	0.267/0.356
Power_Dorsal_Alpha	-0.026/0.928	-0.069/0.814	-0.081/0.784	-0.085/0.774	-0.141/0.631	-0.008/0.979
Power_Dorsal_Beta	-0.359/0.207	-0.141/0.68	-0.307/0.285	-0.3/0.298	-0.355/0.212	-0.352/0.217
Power_Dorsal_Gamma	0.088/0.764	-0.194/0.506	-0.007/0.981	0.069/0.814	-0.034/0.909	0.144/0.624
Power_Ventral_Theta	0.104/0.724	0.23/0.429	-0.291/0.313	0.233/0.423	-0.143/0.626	0.092/0.753
Power_Ventral_Alpha	-0.049/0.869	0.41/0.145	-0.224/0.441	0.059/0.841	-0.083/0.779	0.032/0.934
Power_Ventral_Beta	-0.172/0.557	0.028/0.925	-0.085/0.771	-0.254/0.382	-0.194/0.505	-0.11/0.707
Power_Ventral_Gamma	0.262/0.365	0.064/0.829	0.028/0.925	0.295/0.307	0.056/0.85	0.237/0.351
Firing rate	0.238/0.412	-.757**/0.002	0.298/0.301	0.12/0.682	0.118/0.687	-0.08/0.787
Asymmetry_Index	0.026/0.928	-0.529/0.052	0.095/0.747	-0.182/0.534	-0.002/0.994	-0.103/0.727
MBI	0.157/0.593	-0.41/0.145	0.189/0.517	0.284/0.325	0.181/0.536	0.028/0.924

Values are presented as rho (ρ)/P value. *Represented a statistical correlation ($P < 0.05$). MBI, modified burst index; H&Y, Hoehn and Yahr stages; UPDRS, Unified Parkinson's Disease Rating Scale.

Table S5 Results of the correlation analysis between MER characteristics and clinical characteristics in the TD group (DBS-on).

MER features	UPDRS III (DBS-on)					
	UPDRS III	Tremor	Rigidity	Bradykinesia	Axial symptoms	Gait
Power_Theta	0.226/0.559	-0.317/0.406	-0.122/0.754	0.565/0.113	0.441/0.235	-0.091/0.815
Power_Alpha	0.075/0.847	-0.386/0.305	-0.122/0.754	0.414/0.269	0.373/0.323	-0.091/0.815
Power_Beta	-0.184/0.635	-0.188/0.628	-0.507/0.163	-0.008/0.983	0.373/0.323	-0.183/0.638
Power_Gamma	-0.444/0.232	-0.248/0.521	-0.551/0.124	-0.262/0.496	0.119/0.761	-0.456/0.217
Power_Dorsal_Theta	0.075/0.847	-0.386/0.305	-0.122/0.754	0.414/0.269	0.373/0.323	-0.091/0.815
Power_Dorsal_Alpha	0.159/0.683	-0.297/0.438	-0.122/0.754	0.489/0.181	0.373/0.323	-0.091/0.815
Power_Dorsal_Beta	-0.31/0.417	-0.03/0.94	-0.665/0.051	-0.228/0.555	0.136/0.728	-0.456/0.217
Power_Dorsal_Gamma	-0.017/0.966	0.119/0.761	-0.481/0.19	-0.068/0.863	0.39/0.3	-0.183/0.638
Power_Ventral_Theta	0.318/0.404	-0.129/0.741	-0.14/0.72	0.608/0.083	0.458/0.215	-0.091/0.815
Power_Ventral_Alpha	0.134/0.731	-0.574/0.106	0.14/0.72	0.489/0.181	0.424/0.256	0.091/0.815
Power_Ventral_Beta	0.393/0.295	-0.297/0.438	0.07/0.858	0.439/0.237	**.763/0.017***	0.548/0.127
Power_Ventral_Gamma	-0.251/0.515	-0.188/0.628	-0.393/0.295	-0.152/0.696	0.22/0.569	-0.183/0.638
Firing rate	-0.109/0.781	0.149/0.703	-0.411/0.272	-0.245/0.526	0.153/0.695	0.274/0.476
Asymmetry_Index	0.51/0.16	0.634/0.067	-0.114/0.771	0.279/0.468	0.39/0.3	0.456/0.217
MBI	0.184/0.635	0.129/0.741	0.262/0.495	0.228/0.555	-0.051/0.897	0.091/0.815

Values are presented as rho (ρ)/*P* value. *Represented a statistical correlation ($P < 0.05$). MBI, modified burst index; H&Y, Hoehn and Yahr stages; UPDRS, Unified Parkinson's Disease Rating Scale.

Table S6 Results of correlation analysis between dorsal beta PSD and UPDRS part III in the PIGD group.

**UPDRS Part III**	**Power_Dorsal_Beta**		
	**rho**	***p***	***p*(FDR-Corrected)**
**med-off**			
UPDRS III	-0.273	0.401	0.057
Tremor	0.297	0.345	0.058
Rigidity	0.178	0.603	0.068
Bradykinesia	-0.315	0.272	0.066
Axial symptoms	-0.433	0.122	0.069
Gait	-0.214	0.464	0.060
**med-on**			
UPDRS III	-0.081	0.782	0.078
Tremor	0.153	0.602	0.073
Rigidity	-0.022	0.94	0.088
Bradykinesia	-0.247	0.394	0.061
Axial symptoms	-0.582	**0.029***	**0.049***
Gait	-0.506	0.065	0.055
**DBS-on**			
UPDRS III	-0.359	0.207	0.088
Tremor	-0.141	0.68	0.072
Rigidity	-0.307	0.285	0.060
Bradykinesia	-0.3	0.298	0.056
Axial symptoms	-0.355	0.212	0.072
Gait	-0.352	0.217	0.061

*Represented a statistical correlation ($P < 0.05$). UPDRS, Unified Parkinson's Disease Rating Scale, Power_Dorsal_Beta, the power spectral density of the dorsal beta band, FDR = false discovery rate

Table S7 Results of correlation analysis between dorsal beta PSD and UPDRS part III in the TD group.

**UPDRS Part III**	**Power_Dorsal_Beta**		
	**rho**	***p***	***p*(FDR-Corrected)**
**med-off**			
UPDRS III	-0.678	**0.045***	0.073
Tremor	-0.075	0.847	0.551
Rigidity	0.545	0.129	0.140
Bradykinesia	-0.77	**0.015***	**0.049***
Axial symptoms	0.009	0.953	0.517
Gait	-0.284	0.458	0.373
**med-on**			
UPDRS III	-0.717	**0.03***	**0.029***
Tremor	-0.261	0.498	0.193
Rigidity	-0.603	0.086	0.056
Bradykinesia	-0.828	**0.006***	**0.012***
Axial symptoms	0.451	0.223	0.108
Gait	0.26	0.5	0.162
**DBS-on**			
UPDRS III	-0.31	0.417	0.200
Tremor	-0.03	0.94	0.225
Rigidity	-0.665	0.051	0.073
Bradykinesia	-0.228	0.555	0.200
Axial symptoms	0.136	0.728	0.209
Gait	-0.456	0.217	0.156

*Represented a statistical correlation ($P < 0.05$). UPDRS, Unified Parkinson's Disease Rating Scale, Power_Dorsal_Beta, the power spectral density of the dorsal beta band, FDR = false discovery rate
